# HLBT-100: a highly potent anti-cancer flavanone from *Tillandsia recurvata* (L.) L.

**DOI:** 10.1186/s12935-017-0404-z

**Published:** 2017-03-07

**Authors:** Henry I. C. Lowe, Ngeh J. Toyang, Charah T. Watson, Kenneth N. Ayeah, Joseph Bryant

**Affiliations:** 1 Bio-Tech R&D Institute, University of the West Indies, 6 St. Johns Close, Mona, Jamaica; 2 Educational & Scientific LLC, 725 W Lombard St, Baltimore, MD 21201 USA; 3 Institute of Human Virology, University of Maryland School of Medicine, 725 W Lombard St, Baltimore, MD USA

**Keywords:** *Tillandsia recurvata*, Flavanone, Anticancer, Angiogenesis, Ball Moss

## Abstract

**Background:**

The incidence and mortalities from cancers remain on the rise worldwide. Despite significant efforts to discover and develop novel anticancer agents, many cancers remain in the unmet need category. As such, efforts to discover and develop new and more effective and less toxic agents against cancer remain a top global priority. Our drug discovery approach is natural products based with a focus on plants. *Tillandsia recurvata* (L.) L. is one of the plants selected by our research team for further studies based on previous bioactivity findings on the anticancer activity of this plant.

**Methods:**

The plant biomass was extracted using supercritical fluid extraction technology with CO_2_ as the mobile phase. Bioactivity guided isolation was achieved by use of chromatographic technics combined with anti-proliferative assays to determine the active fraction and subsequently the pure compound. Following in house screening, the identified molecule was submitted to the US National Cancer Institute for screening on the NCI60 cell line panel using standard protocols. Effect of HLBT-100 on apoptosis, caspase 3/7, cell cycle and DNA fragmentation were assessed using standard protocols. Antiangiogenic activity was carried out using the ex vivo rat aortic ring assay.

**Results:**

A flavonoid of the flavanone class was isolated from *T. recurvata* (L.) L. with potent anticancer activity. The molecule was code named as HLBT-100 (also referred to as HLBT-001). The compound inhibited brain cancer (U87 MG), breast cancer (MDA-MB231), leukemia (MV4-11), melanoma (A375), and neuroblastoma (IMR-32) with IC_50_ concentrations of 0.054, 0.030, 0.024, 0.003 and 0.05 µM, respectively. The molecule also exhibited broad anticancer activity in the NCI60 panel inhibiting especially hematological, colon, CNS, melanoma, ovarian, breast and prostate cancers. Twenty-three of the NCI60 cell lines were inhibited with GI_50_ values <0.100 µM. In terms of potential mechanisms of action, the molecule demonstrated effect on the cell cycle as evidenced by the accumulation of cells with <G1 DNA content, activation of caspase 3/7, DNA fragmentation and culminating in apoptotic cell death. HLBT-100 also demonstrated antiangiogenic potential by inhibiting capillary sprout and tube formation in a dose dependent manner in the ex vivo rat aortic ring.

**Conclusion:**

This paper describes for the first time the anticancer activity of HLBT-100 isolated from *T. recurvate* (L.) L. The broad and selective anticancer activity of HLBT-100 as evidenced by its potent activity against IMR-32, CNS cancer cell line while not active against neuro-2a, a normal CNS cell line. The activity demonstrated by HLBT-100 in these studies makes the molecule a potential candidate for further development targeting especially those cancers that remain in the unmet need category such as glioblastoma multiforme and acute myeloid leukemia in addition to other cancers.

## Background

According to recent statistics from the National Cancer Institute (NCI) and the World Health Organization (WHO), there will be about 14.1 million new cancer cases diagnosed globally in 2016 out of which about 1.7 million cases will be in the United States of America (USA) [1–3]. Expenditure associated with cancer care in USA was about US$125 billion in 2010 and it is estimated to reach US$156 billion by 2020 [3]. Cancer as such remains a major worldwide health problem and one of the top causes of death [4]. The search for new, effective and less toxic anticancer drugs as such remains a major global priority. The US government for example has declared cancer a major health problem and is now increasing funding for research and development of new cancer treatments through the newly established Cancer Moonshot project which aims to enhance early detection and prevention of cancer including expanding research in the area of immuno-oncology [5].

For several decades, the world depended on nature as a source of new biologically active molecules as evidenced by the discovery of drugs such as penicillin, quinine, Taxol, vinca alkaloids, metformin and several others [6]. Natural products especially from plants have made significant contributions to the discovery and development of new drugs particularly against cancer [7]. Given the previous success recorded in drug discovery from nature, we decided to base our drug discovery effort on plants given the rich diversity of chemical structures that exist in plants as secondary metabolites [8, 9].

*Tillandsia recurvata* L. (Bromeliaceae) also known as the Jamaican Ball Moss or the Old Man’s beard is an aerial plant often found growing on tree branches or on telephone and electricity poles or cables [10]. Plants from the Bromeliaceae family have a unique capability to survive harsh drought conditions but never seem to die as the leaves come back to life within hours of rehydration and as such, these plants are also known as the “resurrection plants” [11]. Previous studies by our group found *T. recurvata* to be having various chemical constituents including terpenoids and flavonoids [12, 13]. Screening of the extracts of *T. recurvata* also revealed that the plant possesses several pharmacological properties including, anticancer activity [14, 15]. Some of the specific compounds previously isolated from the plant includes cycloartanes, cinnamoyl diccinamates and phenolic compounds [12, 13, 15]. Anti-angiogenic activity and kinase inhibition are some of the specific mechanisms of action demonstrated by the extracts or compounds isolated from *T. recurvata* [16–18]. Based on the activity demonstrated by the crude extract of *T. recurvata*, this study was carried out in an attempt to further isolate and characterize the anticancer molecules present in the plant.

In this study, HLBT-100 isolated from *T. recurvata* has been identified to be a flavonoid with hitherto unreported anticancer activity. Flavonoids are widely distributed in the plant kingdom with over 4000 structures described making flavonoids the largest class of plant secondary metabolites [19, 20]. Flavonoids have a characteristic color ranging from pale to deep orange and their basic backbone structure includes two fused rings linked to an aromatic ring [21–23]. Flavonoids are sub-divided into six classes including flavanols, flavonols, flavones, flavanones, isoflavones and anthocyanidins [24, 25]. In plants, the primary significance of flavonoids is to provided pigmentation required for UV-radiation protection as well as defense from pests and disease [26–28]. In the human sphere, flavonoids provide a wide range of benefits including use in diet and health [20] and are credited for possessing antimicrobial, antiviral, cancer chemoprevention, neurodegenerative properties and cardiovascular health benefits among several other health properties [29–33]. Flavonoids as such hold promise as potential cures for mankind and require increased scientific attention.

## Results

### Chemical characterization of HLBT-100

**Fig. 1:**
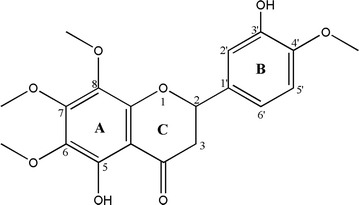
Chemical structure of HLBT-100

Using spectroscopic analysis including mass spectrometry (MS) and nuclear magnetic resonance (NMR) studies, the chemical structure of the yellowish powder obtained was elucidated. The molecule was putatively code named HLBT-001 (5,3′-dihydroxy-6,7,8,4′-tetramethoxyflavanone) and was renamed HLBT-100 once the structure elucidation was completed (Fig. 1). A thorough search of the literature revealed that the compound had previously been isolated from *T. recurvata* [12] but no bioactivity studies were carried out or reported for the molecule. HLBT-100 belongs to the flavanone class of flavonoids which is characterized by a single bond between C2 and C3 of the heterocycle ring “C” compared to the closely related flavonol and flavone classes of flavonoids (Fig. 1). HLBT-100 is highly polymethoxylated and most of the polymethoxylated flavanones have previously been isolated mostly from citrus plants of the Rutaceae family [34, 35].

### Antiproliferative activity

**Fig. 2:**
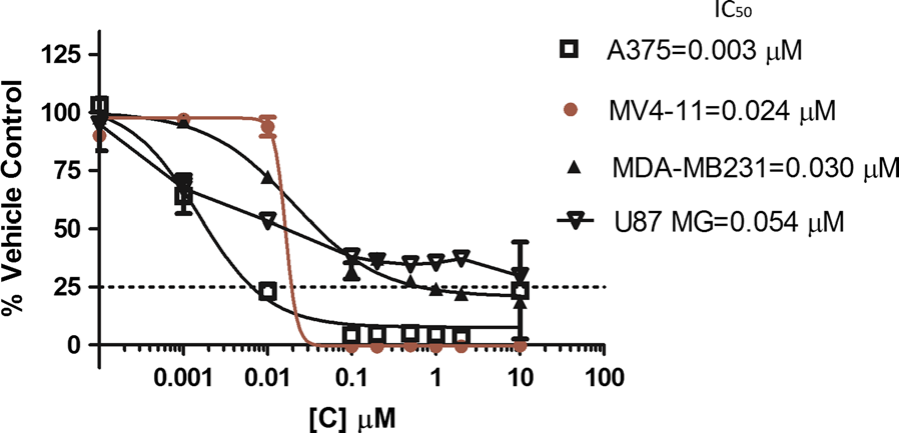
WST-1 assay results for HLBT-100 in 72 h against MV4-11, MDA-MB231. PC-3, A375 and U87 MG. Following treatment of cells, dose response curves were plotted and IC_50_ values calculated using graphpad prism software. The mean IC50s and standard deviations generated are as follows: MV4-11 = 0.024 ± 0.001 µM; MDA-MB231 = 0.030 ± 0.008 µM; PC-3 = 0.031 ± 0.011 µM; A375 = 0.004 ± 0.003 µM; U87 MG = 0.054 ± 0.006 µM

**Fig. 3:**
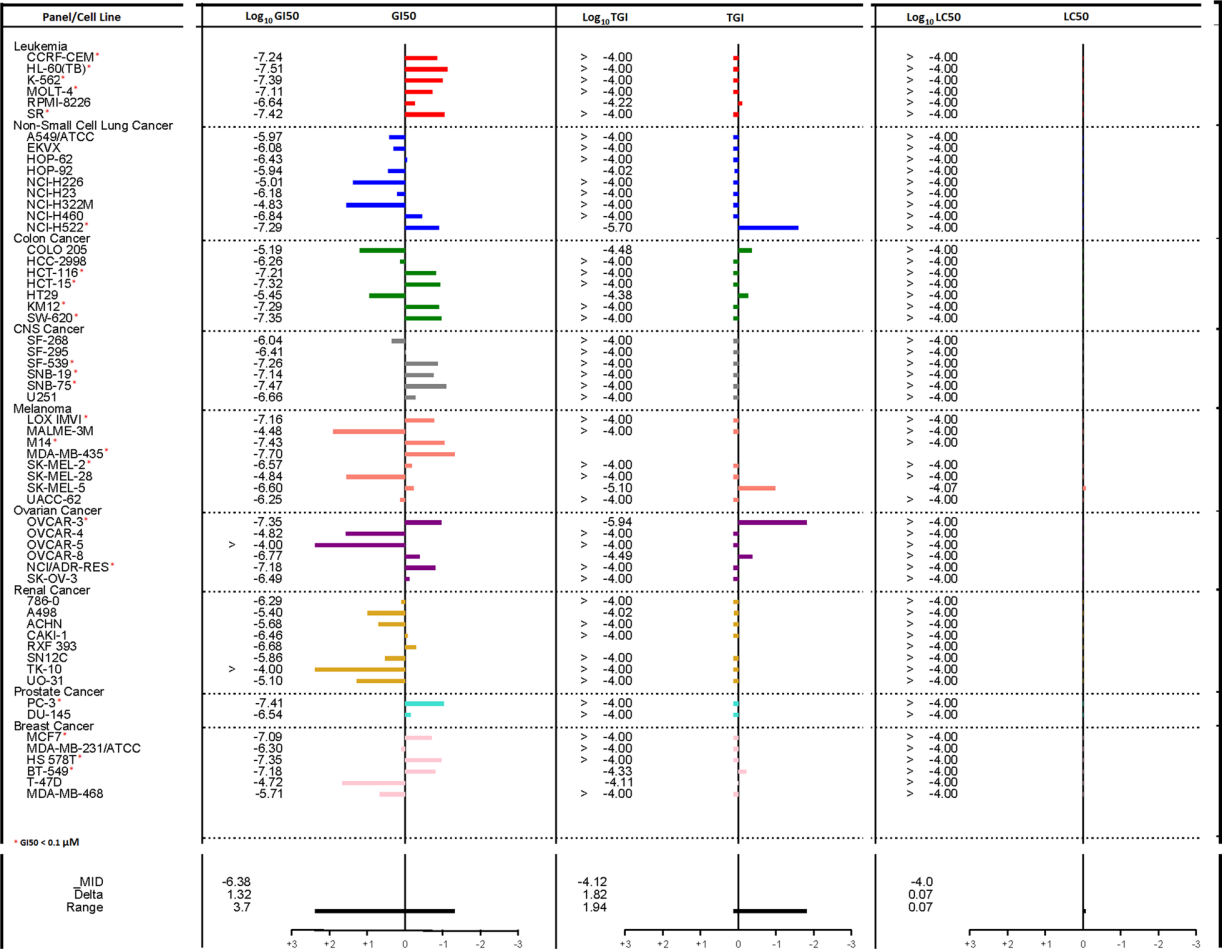
Anticancer activity of HLBT-100 on the NCI60 cancer cell line panel. The compound was first screened at a single concentration of 100 µM and having satisfied the NCI predetermined threshold (data not shown), the molecule was subjected 5-dose response screen

To determine the antiproliferative activity of HLBT-100 against cancer cell lines, the brain cancer cell line (U87 MG), breast cancer cell line (MDA-MB-231), human melanoma (A375), leukemia (MV4-11) and prostate cancer (PC-3) cell lines were subjected to the WST-1 antiproliferative assay. HLBT-100 in a dose dependent manner inhibited the proliferation of cancer cells at sub-micromolar concentrations with IC_50_ values ranging from 0.004 to 0.054 µM (Fig. 2). Upon being subjected to anticancer activity screen on the NCI60 panel, HLBT-100 demonstrated potent activity against 23 of the cell 60 lines on the NCI cancer cell line panel with GI_50_ values <0.100 µM (Fig. 3).

### Cell viability assay

**Fig. 4:**
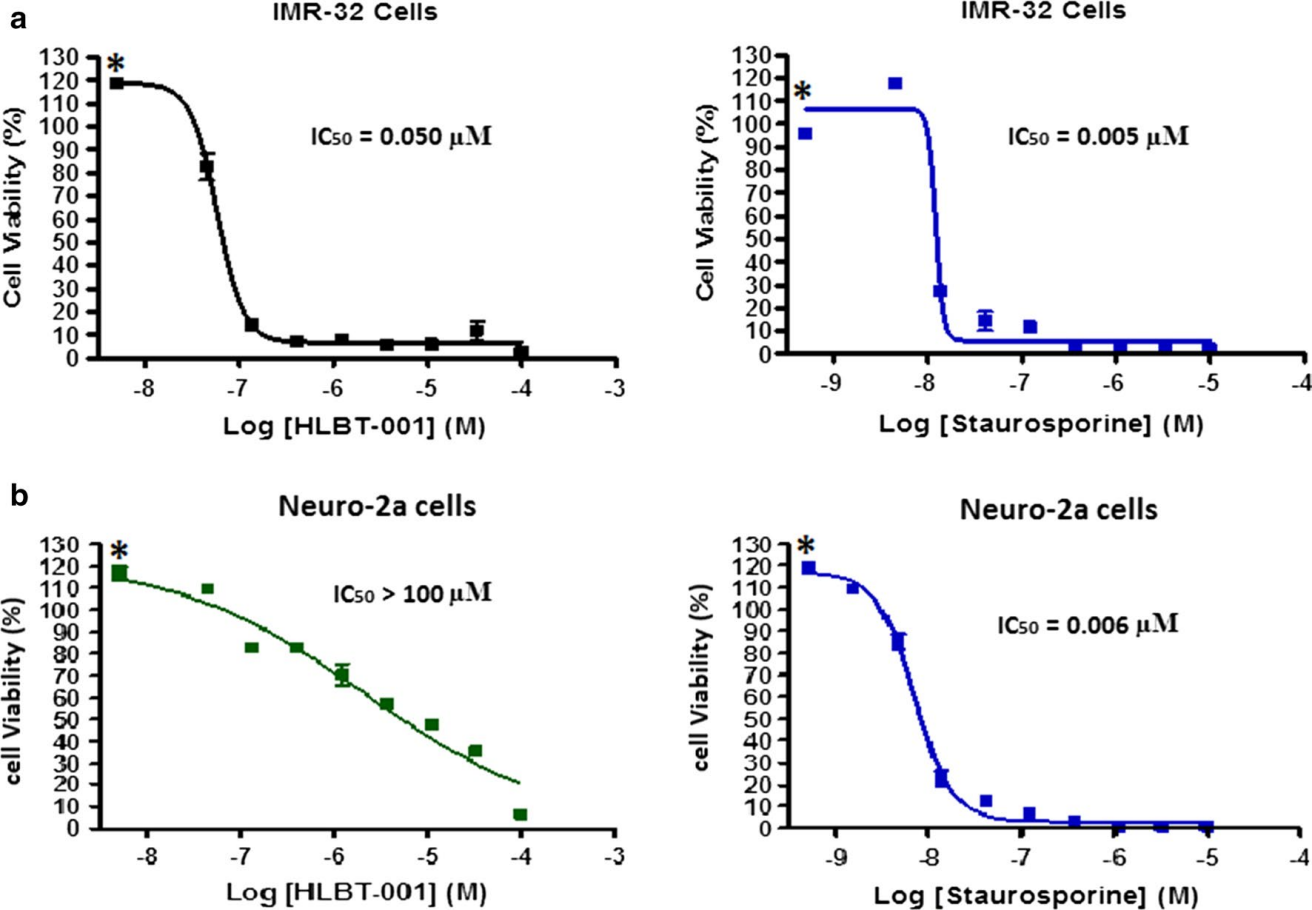
Effect of HLBT-100 on cell viability. Cells were seeded at 2000–5000 cells/well in 96-well plates. After an overnight incubation, cells were treated with HLBT-100 **a** IMR-32 nueroblastoma cancer cell line and **b** neuroblastoma normal cells, and incubated for 72 h at 37 °C. The cell viability was determined using the Cell Titer-Glo luminescent cell viability assay kit after staining for 10 min, according to the manufacturer’s instructions. The IC_50_ values were generated using graphpad prism. Staurosporine (*graphs on the right*) was used as a positive control against both cell lines. *Asterisk* Lower concentrations of HLBT-100 versus control are slightly above 100% of control because of “edge effect” where slight evaporation of media in control wells on the edge of plate might result in lower OD values for those wells

To assess the cell viability and selectivity effect of HLBT-100, the Cell Titer-Glo luminescent cell viability assay kit was used. The neuroblastoma cancer cell line (IMR-32) and normal neuroblastoma cells (nuero-2a) were subjected to this assay. The compound showed great selectivity by killing the cancer cells (IMR-32) while having no effect against the normal human cell line (nuero-2a) (Fig. 4).

### Cell cycle arrest and apoptosis

**Fig. 5:**
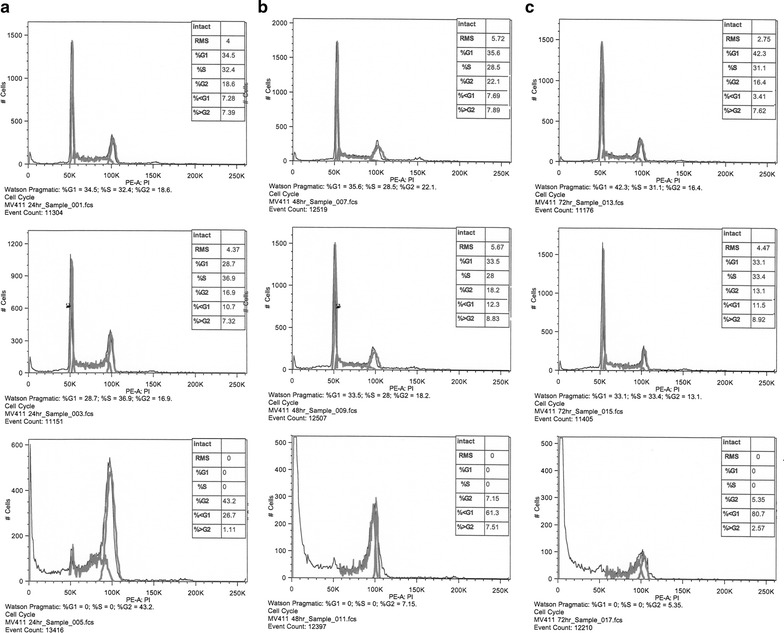
Cell cycle effect of HLBT-100. For cell cycle effect determination, MV4-11 leukemic cell line was used. Cells were treated with either DMSO: *top row* (vehicle) or HLBT-100: *middle row* (0.024 µM) and *bottom row* (0.120 µM). Cell were incubated at 37 °C and at 24 h (**a**), 48 h (**b**) and 72 h (**c**), cells were harvested, washed and stained according to the manufacturer’s recommendations with the Abcam propidium iodide flow cytometry kit. Acquired samples were analyzed using FlowJo (Tree Star, Ashland, OR, USA)

**Fig. 6:**
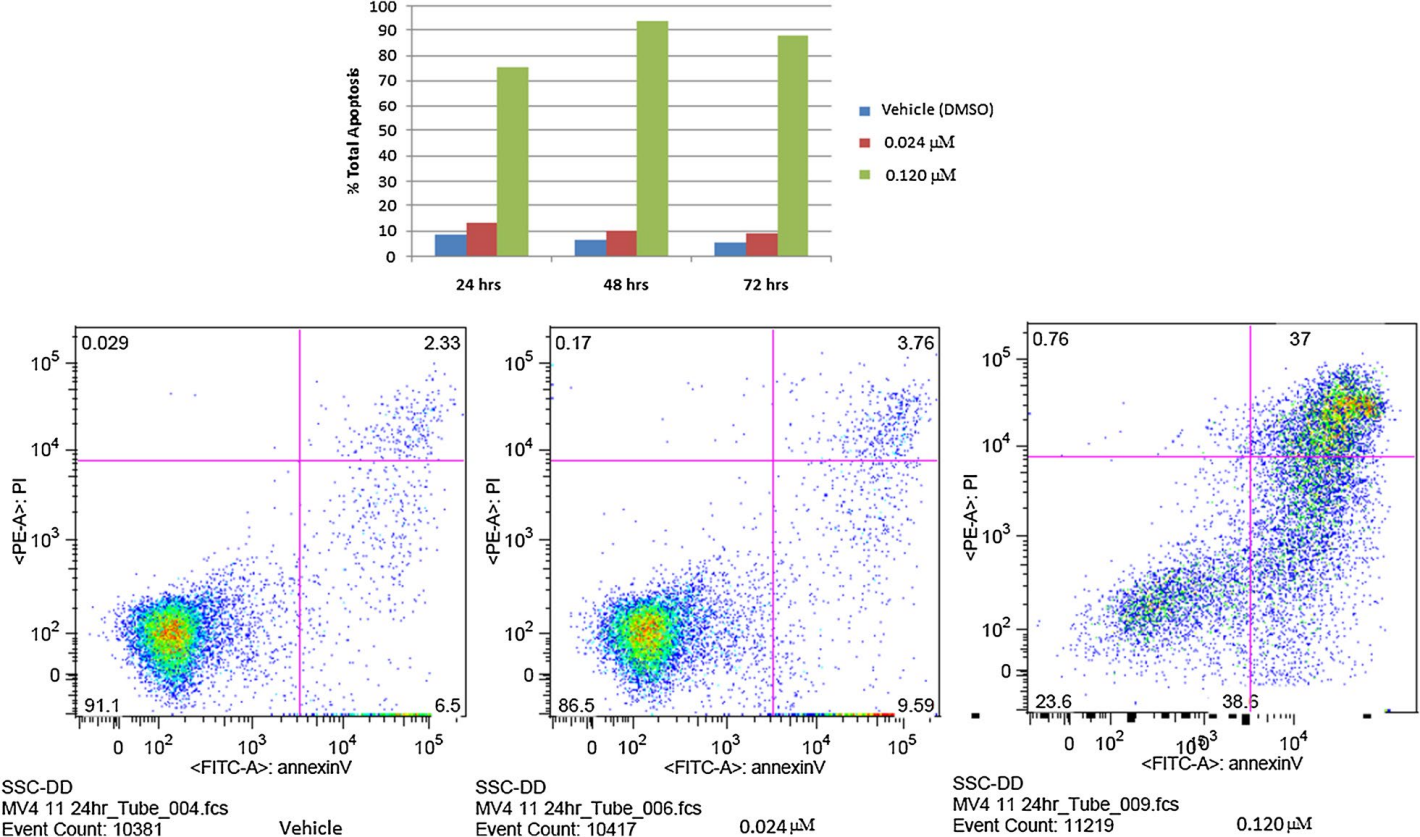
Effect of HLBT-100 on apoptosis. Apoptotic effect of HLBT-100 was assessed on the MV4-11 leukemic cell line using the Annexin V-FITC apoptosis detection Kit I. After treatment with HLBT-100 (0.024 and 0.120 µM) cells were incubated at 37 °C for 24 h. Cells were harvested and stained with Annexin V-FITC and Propidium Iodide reagents. Stained samples were analyzed using FlowJo (Tree Star, Ashland, OR, USA) for apoptotic effect

The effect of HLBT-100 on cell cycle arrest and apoptosis was determined using, the propidium iodide flow cytometry kit and the Annexin V-FITC apoptosis detection Kit. Cell cycle significantly affected as accumulation of cells with <G1 DNA content were at 24 h, 48 h and 72 h of incubation at the 5× IC_50_ in the MV4-11 cell line (Fig. 5). In regards to apoptosis effect, HLBT-100 caused significant apoptotic effect (>70%) at 5× of it’s IC_50_ against MV4-11 cells after 24 h (Fig. 6).

### In-cell DNA fragmentation induced by HLBT-100

**Fig. 7:**
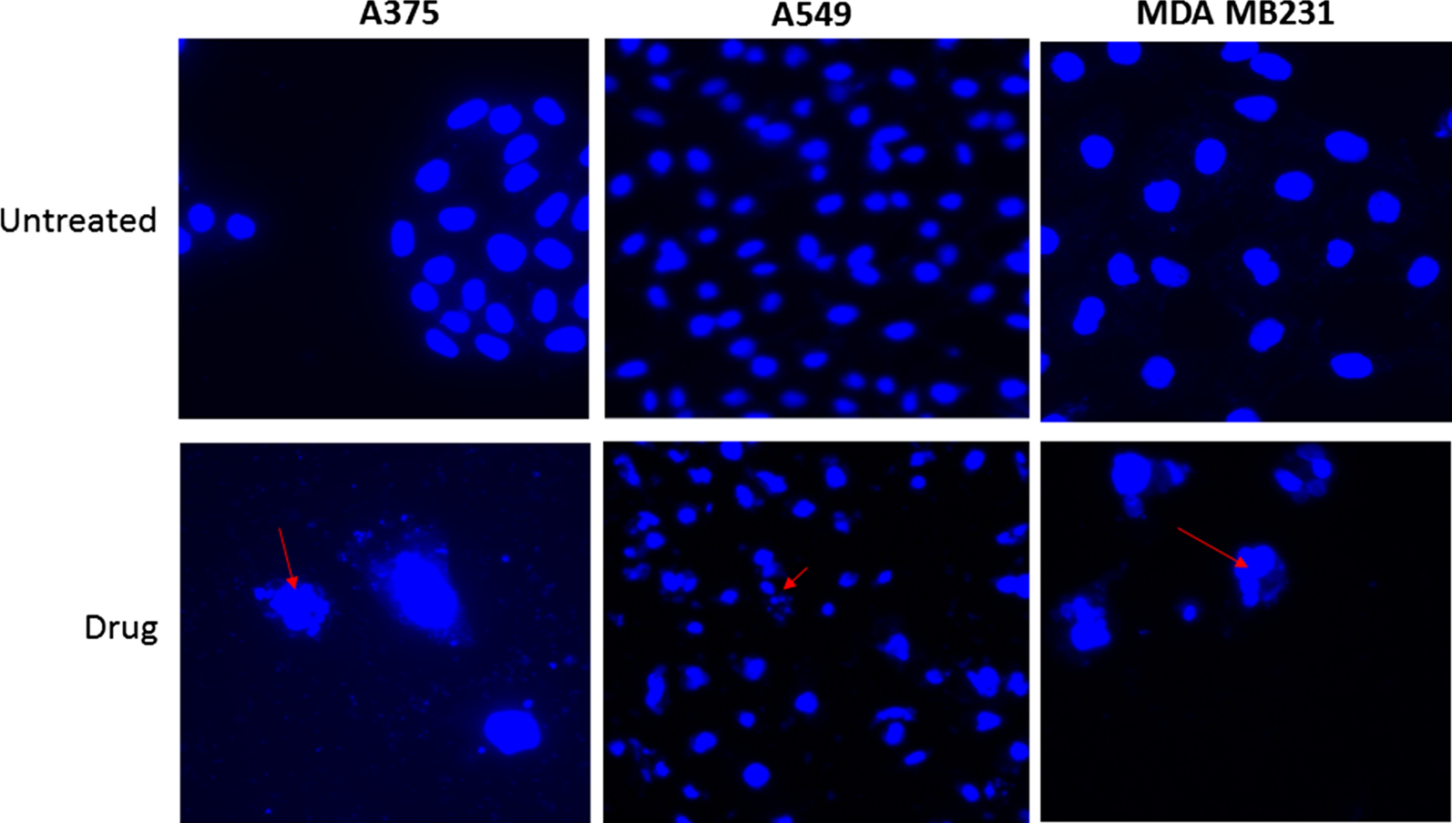
In-cell DNA fragmentation effect of HLBT-100 against the following cell lines: A375, A459 and MDA MB231 was evaluated. 1–2 × 10^5^ cells were seeded on glass coverslip in 12 well cell culture plate (costar, USA) and treated with drug. An equal vol of 10% formalin was added in each well for 10–15 min at RT. The plate was centrifuged at 3000*g* for 20 min to spin down all the cells. Liquid was discarded and cells were treated with 0.1% Triton X100 for 5 min and removed from triton. Cells were treated with DAPI (0.5 μg/ml) in 0.1% BSA for 5 min and washed 3× with normal saline. Finally, the cells were mounted with mowiol (sigma) and transferred onto microscopic glass slides, dried in dark and visualized under fluorescence microscope (Nikon and Zeiss, USA). Effect of HLBT-100 on fragmented is indicated by *arrows*

DAPI nuclear staining showed DNA was fragmented in cells treated with the compound compared to vehicle treated cells (Fig. 7). Maximum cleavage was found with the 72 h treatment with HLBT-100 against all the cell lines tested. Non apoptotic cells showed non-cleaved intact bigger round chromosomal DNA whereas apoptotic cells showed cleaved nuclei.

### Caspase 3/7 activity

**Fig. 8:**
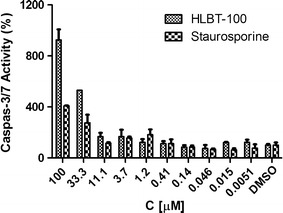
The Apo-ONE^®^ Homogeneous Caspase-3/7 Assay (Promega) was used to detect caspase-3/7 activity of HLBT-100 based on the cleavage of a pro-fluorescent DEVD peptide-rhodamine 110 substrate [(Z-DEVD)2-R110] according to manufacturer’s instruction and as reported by Wagner et al. [78]. PC-3 prostate cancer cell line was cultured in F12 K medium supplemented with 10% FBS and 100 µg/ml penicillin, and 100 µg/ml streptomycin. PC-3 cells were treated with HLBT-100 or reference compound staurosporine for 6 h with serum free medium. The activation was considered significant only when the highest compound dose induced caspase-3/7 activity ≥200% compared to DMSO

To determine if HLBT-100 induces apoptosis via a caspase dependent pathway, the activation effect of HLBT-100 on caspase 3/7 was evaluated. Apoptosis is orchestrated by a family of cysteine proteases known as the caspases. Of the fourteen mammalian caspases identified, caspase 3/7 are thought to coordinate the execution phase of apoptosis by cleaving multiple structural and repair proteins [36]. HLBT-100 upregulated the expression of caspase 3/7 in this study in a dose dependent manner (Fig. 8).

### Angiogenesis

**Fig. 9:**
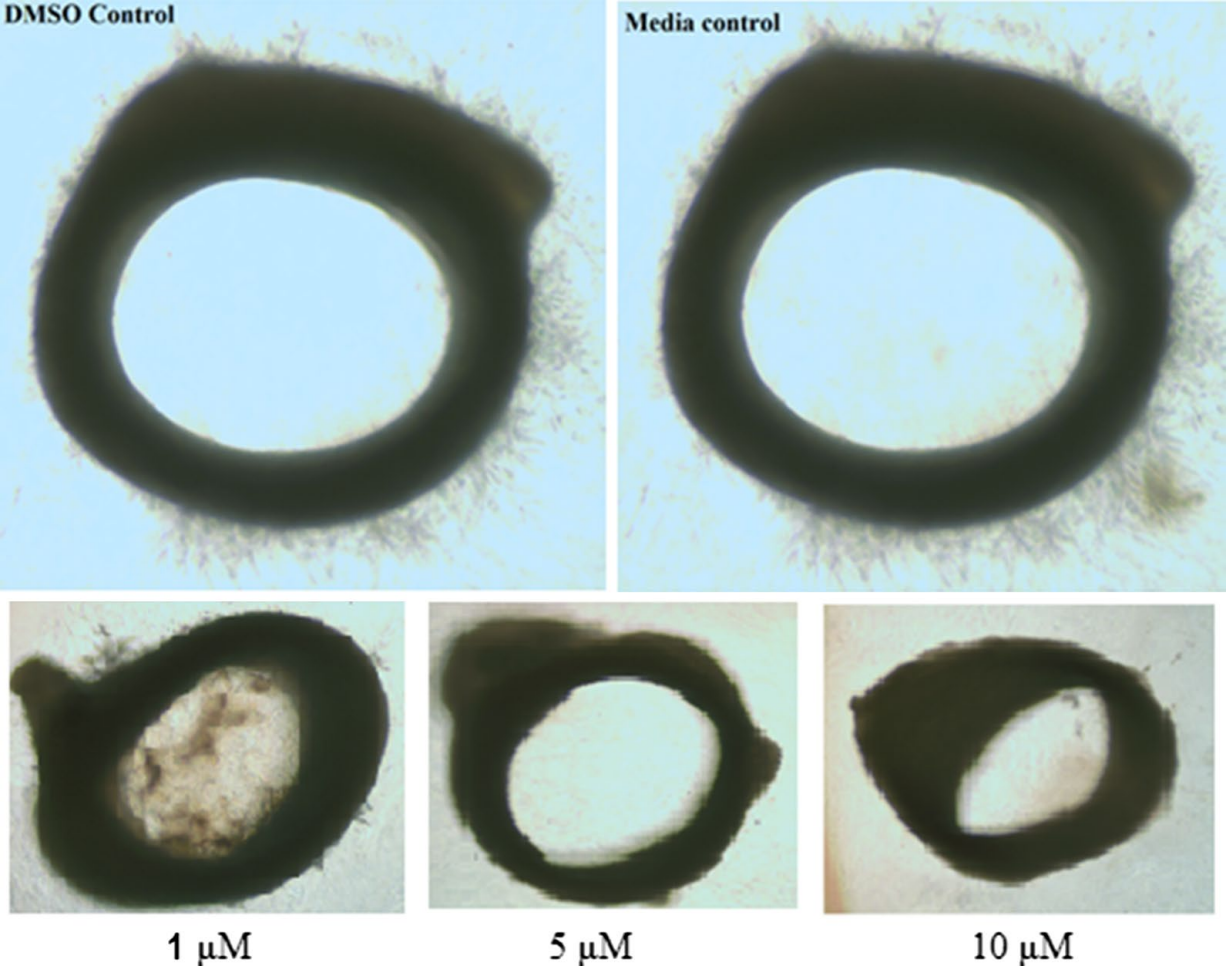
Antiangiogenic activity of HLBT-100. The rat ring aortic assay as was used to determine effect on capillary sprout formation. HLBT-100 inhibited sprout formation at all 3 concentrations tested (1 µM lowest concentration) compared to the DMSO and media controls

The antiangiogenic properties of HLBT-100 were determined using the ex vivo rat aortic ring and in vitro tube formation assays. HLBT-100 demonstrated antiangiogenic activity by inhibiting capillary sprout formation in rat aorta rings (Fig. 9).

## Discussion

The Jamaican Ball Moss continues to demonstrate that it harbors significant bioactive molecules especially against cancer. In this study, we successfully isolated and characterized HLBT-100 from *T. recurvata* as a new anticancer flavonoid (Fig. 1). The potency of HLBT-100 in vitro at sub-micromolar concentrations against both solid and hematological cancers makes HLBT-100 a potential anticancer agent (Figs. 2, 3). HLBT-100 belongs to the flavonoid class of compounds. Flavonoids occur widely in fruits and commonly eaten foods and as such are mostly considered to be safe and as such are used widely for food and disease prevention [30, 37]. Routine consumption of fruits and flavonoid rich foods has long been linked to a lower incidence of several diseases including cancer, neurodegenerative diseases, cardiovascular disease, metabolic disease and immune function modulation [31, 38–44].

HLBT-100 however appears to be a unique molecule as it has not been isolated from any other plant than from *T. recurvata* which is not known to be used as a food plant anywhere [12]. The molecule was identified as a flavanone which has an aglycone that is structurally different from the commonly used flavonoids such as the flavones including compounds such as apeginin, luteolin, kaempferol and quercetin. The major difference between a flavanone and a flavone is the number of bonds between carbons 2 and 3 of the aglycone (Fig. 1). While flavanones have a single bond, flavones have a double bond between carbons 2 and 3. This structural difference seem to confer flavanones with a superior anticancer activity compared to flavanones. For example, Cabrera et al. [45] screened over 50 flavonoids against 3 human cancer cell lines and the most active compounds against the cell lines screened were flavonoid precursors (chalcones) followed by flavanones ahead of the flavones. Even though widely distributed, the flavanones are more commonly found in plants of the citrus family [46, 47].

In the current studies, HLBT-100 demonstrated a dose dependent inhibition of cancer cell lines (Fig. 2). The most sensitive cell line in these studies was the human melanoma cell line (A375) even though activity against the other cancers was also considered to be potent given the sub-micromolar IC_50_ values exhibited. The activity demonstrated against 23 of the 60 cell lines on the NCI60 cancer cell line panel validated the in house anticancer activity demonstrated during the preliminary screen of this molecule (Fig. 3). The NCI60 cancer cell line panel which was established in the 1960s to enhance the discovery and development of novel anticancer agents has been used for several decades as reference tool for cancer drug discovery and is credited for the discovery and development of a number of important anticancer agents in clinical use today [48–50]. The molecule demonstrated selectivity and potently inhibited all the cancer cell lines in the leukemia panel while it was inactive against 7 out of the 8 cell lines in the renal cancer panel. Most importantly, the compound showed the ability to selectively inhibit cancer cells while sparing normal cells as demonstrated in its cytotoxicity towards the neuroblastoma cancer cell line IMR-32 while having no activity against the normal neuro-2a cells (Fig. 4). The neuro-2a cell line is currently viewed as a useful tool to screen novel compounds for potential neurotoxic properties [51]. The lack of toxicity against neurons by HLBT-100 is an early indication that the molecule may possess a very good safety profile.

Apoptosis which is a physiological rather accidental cell death is crucial in the control of the proliferation of cancer cells [52]. While there are a number of mechanisms and molecular events by which apoptosis is elicited, HLBT-100 in this study showed that it activates caspase 3/7, DNA fragmentation and significant effect on cell cycle as (Figs. 5, 7, 8). Caspases are genes that regulate homeostasis through the regulation of cell death and caspase-3, -6 and -7 are considered executioner caspases in the apoptosis process while caspases-2, -8 -9 and -10 are the effector kinases [53, 54]. DNA cleavage is another hallmark of apoptosis and cells treated with HLBT-100 showed significant DNA cleavage following DAPI staining [55, 56]. One of the widely studied flavanones is naringenin which demonstrated GO/G1 G2/M phase arrest in cell cycle studies even though at significantly higher concentrations (>20 µM) compared to HLBT-100 (<0.15 µM) [46, 57]. Most of the commonly used flavonoids have been reported to play a role in cancer chemoprevention through the apoptosis signaling pathway and as such it is not surprising that HLBT-100 exhibits potent apoptosis signaling and at concentrations much less than those exhibited by other commonly used flavonoids [58–60].

Angiogenesis is simply defined as the process by which new vessels are developed and this has significant implication in disease especially tumors which need new vessels for the supply of nutrients [61, 62]. Capillary sprouts as demonstrated by the ex vivo rat aortic ring assay are important in cancer progression as they are known to be representative of all phases of angiogenesis [63]. Several flavonoids including flavopiridol, quercetin, apigenin and naringenin are credited for exhibiting antiangiogenic properties and this property is thought play a role in their antitumor and cancer chemoprevention potential [64–68]. Angiogenesis is also required for cell invasion and migration and as such a necessary conduit for cancer metastasis [66]. Angiogenesis inhibitors are among several anticancer agents that are currently in clinical use and because most of them are kinase inhibitors plagued with resistance issues, there is a need for the discovery and development of new antiangiogenic agents [69, 70]. While the mechanism of action of most antiangiogenic inhibitors involve the inhibition of protein kinases (e.g. VEGFR), the mechanism of inhibition of angiogenesis by HLBT-100 among other mechanism of action studies remain to be determined.

The development of flavonoids into standard drugs remains a challenge despite the plethora of preclinical data that has been reported on their pharmacological properties [20]. Their therapeutic use have been hindered by poor drug-likeness properties to low bioavailability. This attributes accounts for why there are only a handful of flavonoid based molecules currently in clinical trials to-date and includes flavopiridol (alvocidib) (Tolero Pharmaceuticals, Inc.) and Icaritin (Shenogen Pharma, China) [71–73]. To overcome the preclinical development of HLBT-100, a number of approaches including the use of polymer and nanotechnology drug delivery approaches might be required to overcome especially poor bioavailability challenges.

## Conclusion

This paper describes for the first time the anticancer activity of HLBT-100 isolated from *T. recurvata*. The identification of possible anticancer properties including effect on apoptosis and angiogenesis requires further studies to identify the specific molecular and cellular pathways responsible for the observed activity. Further studies are in progress to identify other possible mechanisms of action as well as in vivo efficacy and safety. The broad and selective anticancer activity of HLBT-100 was evidenced by its potent activity against IMR-32, CNS cancer cell line while inactive against neuro-2a, a normal CNS cell line. The activity demonstrated by HLBT-100 in these studies makes the molecule a potential candidate for further development targeting especially those cancers that remain in the unmet need category such as glioblastoma multiforme and acute myeloid leukemia in addition to other cancers neuroblastoma, lymphoma and leukemia.

## Methods

### Compound extraction and isolation

**Fig. 10:**
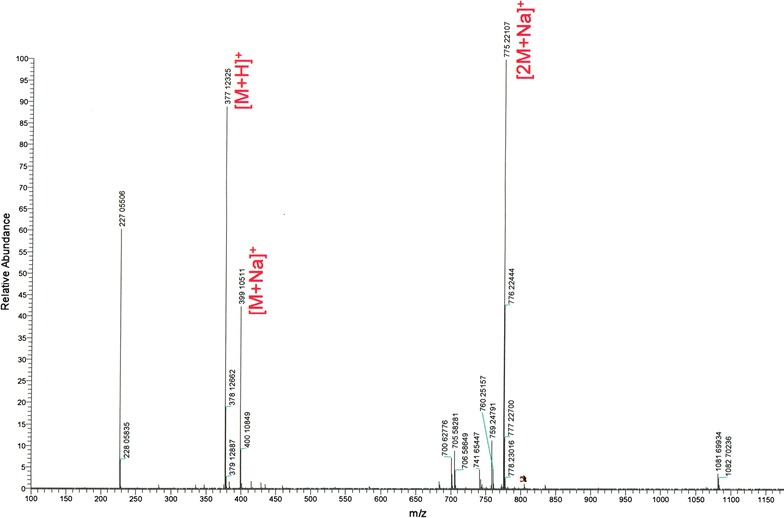
A high resolution mass spectra (HRMS) of HLBT-100. Molecular weight = 376.37 [Mz + H]

The fresh plant material was collected from power lines in Jamaica after which it was air dried under shed away from direct sunlight, then milled and extracted using supercritical fluid extraction (SFE) technology. The extraction parameters on the SFE were set as follows: pressure (200 bars); temperature (heat exchanger −45 °C, extracting vessel −40 °C and collecting vessel −40 °C); solvent (CO_2_ and 10% ethanol); flow rate (9 ml/min) and run time (60 min). Isolation of the bioactive fractions was done in two phases. First, solvent/solvent partition was carried out to separate the lipid soluble from the mid-polar fraction using a combination of methanol, ethyl acetate and DiH_2_O. The two fractions were subjected to bioactivity screening and the mid polar fraction demonstrated significant activity against cancer cell lines. During phase two, advanced separation technology using flash chromatography resulted in the separation of several fractions which were pooled based on thin layer chromatography similarities (TLC) (data not shown). The pooled fractions were subjected to bioactivity screening. The most bioactive fraction was found to have a major compound by TLC which upon further purification yielded a pale yellowish powder and was identified to be a flavanone based on NMR and HRMS analysis (Fig. 10).

### Anti-proliferative assay

#### Cell lines and culture medium

Five human cancer cell lines (U87 MG, MDA-MB-231, MV4-11, PC-3 and A375 were obtained from American Type Culture Collection (ATCC) (Manassas, VA, USA). The five tumor cell lines were maintained in minimum essential media supplemented with 10% fetal calf serum (Thermo Scientific, USA), 1% l-glutamine, 2% penicillin–streptomycin, and 0.2% gentamicin all from Corning Cellgro Mediatech, Inc. (Manassas, USA). The two CNS related cell lines, cancer cell line (-IMR-32) and a normal cell (neuro-2a) were maintained at Reaction Biology Corp.

### Anticancer cell proliferation and cytotoxicity assay

The WST-1 (4-[3-(4-iodophenyl)-2-(4-nitrophenyl)-2H-5-tetrazolio]-1,3-benzene disulfonate) (Roche) colorimetric assay was used [74]. Briefly, cells were trypsinized and plated into 96 well plates in 50 µl of media and incubated overnight. Approximately 18 h after plating, 50 µl of media containing the required drug concentration was added per well. The compounds and extracts were solubilized in DMSO. The cells were allowed to proliferate for 72 h 37 °C in humidified atmosphere of 5% CO_2_. The experiment is terminated using WST-1 (Roche) 10 µl per well and absorbance is read at 450/690 nm. Antiproliferation activity was assessed as a percentage of proliferation of untreated cells, and IC_50_ values determined using Graphpad Prism software (Version 6.01). All concentrations are treated in duplicate and the mean results determined are automatically used in the IC_50_ determination. The anticancer activity against the NCI60 cancer cell lines panel was performed using NCI’s standard protocol [75].

### CellTiter-Glo^®^ luminescent cell viability assay

Cells were seeded at 2000–5000 cells/well in 96-well plates. After an overnight incubation, cells were treated with drug and incubated for 72 h at 37 °C. The cell viability was determined using the Cell Titer-Glo luminescent cell viability assay kit (Promega, Madison, WI, USA) after staining for 10 min, according to the manufacturer’s instructions. The half-maximal inhibitory concentration (IC_50_) values were calculated using Graph Pad Prism software (Version 6.01).

### Cell cycle arrest and apoptosis determination studies

The MV4-11 leukemic cell line was used determine the cell cycle arrest and apoptotic effects of HLBT-100. Cell were treated with either DMSO (vehicle) or HLBT-100. At 24, 48 and 72 h, cells were harvested, washed and stained according to the manufacturer’s recommendations with the Abcam propidium iodide flow cytometry kit (Abcam, Cambridge, MA, USA) for cell cycle determination and the Annexin V-FITC apoptosis detection Kit I for apoptosis assessment (BD Biosciences, San Jose, CA, USA). Samples stained for Annexin V-FITC analysis and Propidium Iodide were acquired using BD FACS Canto II (BD Biosciences, San Jose, CA, USA). Acquired samples were analyzed using FlowJo (Tree Star, Ashland, OR, USA).

### In-cell DNA fragmentation assay

The method used for nuclear DNA fragmentation has been described previously [76, 77] with slight modification. Briefly, 1–2 × 10^5^ cells were seeded on the glass coverslip in 12 well cell culture plate (costar, USA). The cells were treated with the drugs and at the end of treatment equal vol of 10% formalin was added in each well for 10–15 min at RT. The plate was centrifuged at 3000*g* for 20 min to spin down all the cells. The liquid was discarded and cells were treated with 0.1% Triton X100 for 5 min and after that, the triton was removed. In the next step, Cells were treated with DAPI (0.5 μg/ml) in 0.1% BSA for 5 min and washed three times with normal saline. Finally, the cells were mounted with mowiol (sigma) and transferred onto microscopic glass slides, dried in dark and visualized under fluorescence microscope (Nikon and Zeiss, USA).

### Caspase 3/7 activation

The Apo-ONE^®^ Homogeneous Caspase-3/7 Assay (Promega) was used to detect caspase-3/7 activity based on the cleavage of a pro-fluorescent DEVD peptide-rhodamine 110 substrate [(Z-DEVD)2-R110] according to manufacturer’s instruction and as reported by Wagner et al. [78]. PC-3 prostate cancer cell line was cultured in F12K medium supplemented with 10% FBS and 100 µg/ml penicillin, and 100 µg/ml streptomycin. PC-3 cells were treated with HLBT-100 or reference compound staurosporine for 6 h with serum free medium. The activation was considered significant only when the highest compound dose induced caspase-3/7 activity ≥200% compared to DMSO.

### Angiogenesis

The assay was carried out as previous reported with modification to determine the antiangiogenic activity of HLBT-100 [63, 79]. Briefly, a 170 g (5–6 weeks old) Sprague-Dawley rat (Harlan, Frederick, Maryland) was euthanized by CO_2_ asphyxiation. All rats used were maintained in the vivarium at the Institute of Human Virology at the University of Maryland, School of Medicine in accordance with the Institutional Animal Care and Use Committee (IACUC) guidelines. The aorta was dissected using a dissecting microscope and the periaortic fibroadipose tissue was removed and 1–2 mm long aortic rings were sectioned and rinsed extensively in EBM media (endothelial cell basal media). The rings were embedded in 200 µl of matrigel in 24-well culture plates so that the lumen was parallel to the base of the plate. 800 µl of EBM without ECGS was added to each well. The rings were incubated for 24 h in an incubator at 37 °C and 5% CO_2_ in humidified air and the media was replaced with fresh 800 µl of EBM with ECGS having varying concentrations of HLBT-100. The rings were further incubated for 4–5 days and evaluated for sprout formation. Capillary sprout formation compared to control were captured with a Nikon FDX-35 camera mounted onto a Nikon Eclipse TE300 microscope.

### Statistical analysis

All experiments where required were run with replicates and the means used in the analysis and IC_50_ values generated and graphing of data done by Graph Pad Prism software (Version 6.01).

## Abbreviations

ATCC: American Type Culture Collection
Cdk: cyclin dependent kinase
CO_2_: carbon dioxide
DAPI: 4′,6-diamidino-2-phenylindole
DNA: dioxyribonucleic acid
DiH_2_O: distilled water
DMSO: dimethyl sulfoxide
EBM: endothelial basal medium
ECGS: endothelial cell growth supplement
FITC: flourescein
HRMS: high resolution mass spectrometry
HUVEC: human umbilical vein endothelial cell
IACUC: Institutional Animal Care and Use Committee
MS: mass spectrometry
NCI: National Cancer Institute
NMR: nuclear magnetic resonance spectroscopy
SFE: supercritical fluid extraction
TLC: thinlayer chromatography
VEGFR: vascular endothelial growth factor receptor
WHO: World Health Organization
WST-1: water-soluble tetrazolium salts
